# Association Between Before-bedtime Passive Body Heating and Nocturia During the Cold Season Among Older Adults

**DOI:** 10.2188/jea.JE20210471

**Published:** 2023-08-05

**Authors:** Yoshiaki Tai, Kenji Obayashi, Kazuki Okumura, Yuki Yamagami, Hiromitsu Negoro, Norio Kurumatani, Keigo Saeki

**Affiliations:** 1 Department of Epidemiology, Nara Medical University School of Medicine, Nara, Japan; 2 Department of Psychiatry, Nara Medical University School of Medicine, Nara, Japan; 3 Department of Urology, University of Tsukuba, Ibaraki, Japan

**Keywords:** nocturia, lower urinary tract symptoms, cold temperature, baths, passive body heating, hyperthermia

## Abstract

**Background:**

Cold exposure induces lower urinary tract symptoms, including nocturia. Cold-induced detrusor overactivity can be alleviated by increasing skin temperature in rats. However, no study has shown an association between passive heating via hot-water bathing and nocturia among humans.

**Methods:**

We included 1,051 Japanese community-dwelling older adults (mean age: 71.7 years) in this cross-sectional study from 2010 to 2014. The number of nocturnal voids was recorded in a self-administered urination diary. Nocturia was defined as ≥2 nocturnal voids. We evaluated bathing conditions in the participants’ houses.

**Results:**

Hot-water bathing (*n* = 888) was associated with a lower prevalence of nocturia than no bathing (*n* = 163), independent of potential confounders, including age, sex, obesity, income, physical activity, diabetes, medication (diuretics, nondiuretic antihypertensives, and hypnotics), depressive symptoms, indoor/outdoor temperature, and day length (odds ratio [OR] 0.68; 95% confidence interval [CI], 0.48–0.97; *P* = 0.035). Compared with the quartile group with the longest bath-to-bed interval (range: 161–576 min), the second and third quartile groups (range: 61–100 and 101–160 min, respectively) were associated with a lower prevalence of nocturia, after adjusting for water temperature and bathing duration besides the same covariates (OR 0.60; 95% CI, 0.38–0.96; *P* = 0.031 and OR 0.59; 95% CI, 0.37–0.94; *P* = 0.025, respectively).

**Conclusion:**

Hot-water bathing, particularly with a bath-to-bed interval of 61–160 min, was significantly associated with a lower prevalence of nocturia among older adults.

## INTRODUCTION

Nocturia, defined as waking to pass urine during the main sleep period,^1^ is an unpleasant symptom, and ≥2 nocturia episodes are clinically significant.^2^ Up to 59.3% of adults aged ≥70 years experience ≥2 nocturia episodes.^3^ The condition is associated with various morbidities, such as obesity, diabetes, hypertension, cardiac disease, and depression; these morbidities may contribute to a higher risk of mortality.^4^^–^^6^

Cold exposure results in lower urinary tract symptoms (LUTS). Previously, low ambient temperature-induced detrusor overactivity in rats was found to be correlated with decreased skin temperature.^7^ Epidemiological studies have reported that the prevalence of nocturia was high in winter and in indoor environments with low temperatures.^8^^,^^9^

Passive body heating (PBH) can alleviate cold stress,^10^ and is associated with the causative factors of nocturia. Previous studies have shown that hot-water bathing with optimal timing is associated with a higher skin temperature at bedtime, higher sleep efficiency, shorter sleep onset latency, and lower night-time blood pressure.^11^^–^^13^ Moreover, high-frequency hot-water bathing was correlated with a lower B-type natriuretic peptide level.^14^ A randomized crossover study involving seven female inpatients aged ≥65 years found no significant effect of foot bathing on night-time urinary frequency, but the sample size may have been insufficient.^15^ In a previous randomized controlled trial (RCT), an eight-item behavioral therapy, including taking a bath, was associated with a reduced number of nocturnal voids, although the achievement rate of taking a bath was only 41%.^16^

Although hot-water bathing before bedtime is a prevailing practice in Japan, particularly among older adults, and is related to the causative factors of nocturia, to our knowledge, no study has reported an association between PBH with hot-water bathing and nocturia. Hence, the present study was conducted to investigate the association between PBH via hot-water bathing and nocturia among community-dwelling older adults. The association between bathing conditions and nocturia was also evaluated.

## METHODS

### Participants and study protocol

This cross-sectional analysis was performed using baseline data from the HEIJO-KYO cohort.^17^ This cohort study included 1,127 community-dwelling older individuals aged ≥60 years who were voluntarily assembled with the cooperation of local associations of older adults. The baseline survey was conducted between September 2010 and April 2014. The 1,122 participants were assessed during the cold season (from October to April). We excluded 16 and 55 participants who did not complete a self-administered urination diary and the measurements of bathing conditions, respectively. Therefore, a total of 1,051 participants were assessed. The study protocol has been described in our previous study.^17^ In summary, we visited each participant’s residence and assessed the indoor environment and their behavior, including bathing, for 48 consecutive hours from the noon of the first day. Nocturnal urination was evaluated on the second night. After receiving sufficient explanation regarding the study, all participants gave their written informed consent. This study was conducted in accordance with the principles of the Declaration of Helsinki and approved by the ethics committee of Nara Medical University (no. 301).

### Night-time frequency and urine volume

The number of nocturnal voids except for the last void before bedtime and the first void after rising time (night-time frequency) was recorded for one night in the self-administered urination diary. Nocturia was defined as ≥2 nocturnal voids. Each participant collected urine samples in a bottle during night-time, which included the first morning void (nocturnal urine volume). After retrieving the bottle, we measured and recorded the total volume. The nocturnal urine production rate (mL/hour) was calculated as nocturnal urine volume divided by time spent in bed. Single voided urine volume was calculated as nocturnal urine volume divided by the number of nocturnal voids including the first morning void.

### Bathing conditions and indoor temperature

The presence or absence of hot-water bathing, which was defined as taking a bathtub bath (excluding taking a shower), was recorded in a self-administered bathing diary. The start and end time of bathing, time in the bathtub (bathing duration), bedtime, and rising time were logged in the diary. The interval from the start time of bathing to bedtime (bath-to-bed interval) was calculated as bedtime minus the start time of bathing. The bathtub water temperature was measured using a logger (Thermochron iButton; Maxim Integrated, Dallas, TX, USA) at 1-minute intervals. The bathroom, dressing room, and living room temperatures were measured 60 cm above the floor at 10-minute intervals using the same logger, which we placed in each participants’ home during the consecutive 48-hour session. We calculated the mean temperature of the bathtub water (hot-water temperature), bathroom, and dressing room while the participants were in the bathroom, according to the record. Because of technical errors, we excluded some data on hot-water temperature using the same methods previously reported.^12^ Daytime indoor temperature was defined as the mean living room temperature between rising time and bedtime of the second day.

### Other measurements

Body mass index (BMI) was calculated as body weight (kg) divided by height (m^2^). The presence of obesity was defined as BMI ≥25 kg/m^2^. Current smoking and drinking habits, household income, medication use, and medical history were reported using a self-administered questionnaire and were confirmed via a medical interview. Diabetes mellitus was determined based on medication use, medical history, or glycated hemoglobin ≥6.5%. Estimated glomerular filtration rate (eGFR) was calculated using the estimating equation for the Japanese population.^18^ Physical activity level was assessed using the Japanese version of the International Physical Activity Questionnaire.^19^^,^^20^ Participants with depressive symptoms were defined as participants with a score higher than five on the 15-item Geriatric Depression Scale (GDS).^21^ Day length (between sunrise and sunset) in Nara on the measurement day was extracted using data from the National Astronomical Observatory of Japan. Outdoor temperature at 10-minute intervals was estimated using data from the local meteorological office in Nara (latitude, 34°N). Daytime outdoor temperature was defined as the mean outdoor temperature between rising time and bedtime.

### Statistical analysis

Normally distributed variables were presented as mean and standard deviation (SD), and non-normally distributed variables as median and interquartile ranges. Means, medians, and proportions were compared using the unpaired *t*-test, Mann–Whitney U test, and χ^2^ test, respectively. Single voided urine volume was determined after natural log-transformation because of its non-normal distribution. Trends in the association between night-time frequency and bathing/sleep variables were evaluated using the linear regression model.

Logistic regression models were used to calculate the odds ratio (OR) of nocturia between the bathing and no bathing groups. The OR was adjusted for potential confounders, including basic variables (age [years], sex, obesity [BMI ≥25 kg/m^2^], current smoking status, alcohol consumption [≥30 vs <30 g/day], household income [≥4 vs <4 million Japanese yen]), clinical variables (use of diuretics, nondiuretic antihypertensives, hypnotics, depressive symptoms [15-item GDS ≥6], diabetes mellitus, eGFR [≥60 vs <60 mL/min/1.73 m^2^], physical activity [per quartile change]), and environmental variables (daytime indoor temperature [°C], daytime outdoor temperatures [°C], daylength [per quartile change]). The difference in log-transformed single voided urine volume between the bathing and no bathing group was calculated using the linear regression model adjusted for basic, clinical, and environmental variables.

To evaluate the associations of bath-to-bed interval, bathing duration, and hot-water temperature with nocturia, we performed a subgroup analysis of participants who bathed. They were divided into four groups according to the quartile values of the bath-to-bed interval due to its non-normal distribution. The ORs of nocturia between the Q1, Q2, and Q3 groups and the Q4 group of bath-to-bed interval were calculated because the Q4 group may have had the lowest heat load remaining at bedtime. The ORs were adjusted for bathing variables, bedtime (clock time), and time in bed (hours), in addition to all covariates used in the analysis between the bathing and no bathing groups. The Poisson regression models with generalized estimating equations were used to evaluate the association of the three bathing variables with night-time frequency by calculating the ratios of night-time frequency (ratios of the Q1/Q4, Q2/Q4, and Q3/Q4 groups of the bath-to-bed interval). The ratios were adjusted for the same covariates used in the logistic regression models in the subgroup analysis.

All analyses were performed using SPSS for Windows (version 26.0; IBM SPSS Inc., Chicago, IL, USA). All *P*-values were two-sided, and a *P*-value of <0.05 was considered statistically significant.

## RESULTS

### Baseline demographic and clinical characteristics

Of 1,051 participants (mean age: 71.7; SD, 7.1 years), 562 (53.5%) were women, 888 (84.5%) bathed, and 308 (29.3%) experienced nocturia. Significant differences were found in age, day length, and single voided urine volume; proportions of obesity and household income (>4 million Japanese yen); and prevalence of nocturia between the bathing and no bathing groups (Table 1). By contrast, there were no significant differences in the nocturnal urine volume or nocturnal urine production rate between the two groups.

**Table 1.** Basic, clinical, and environmental characteristics of participants (*n* = 1,051)

	Hot-water bathing		*P*

	without (*n* = 163)	with (*n* = 888)	
Basic variables			
Age, years, mean (SD)	74.6 (7.3)	71.2 (6.9)	<0.001
Sex, female, *n* (%)	93 (57.1)	469 (52.8)	0.318
Body mass index, kg/m^2^, mean (SD)	22.6 (3.1)	23.2 (3.0)	0.029
Current smoker, *n* (%)	9 (5.5)	43 (4.8)	0.713
Alcohol consumption, ≥30 g/day, *n* (%)	16 (9.8)	135 (15.2)	0.072
Household income, ≥4 million JPY/year, *n* (%)	48 (31.4)	379 (46.2)	0.001
Clinical variables			
Diabetes mellitus, *n* (%)	22 (13.6)	98 (11.2)	0.387
eGFR <60 mL/min/1.73 m^2^, *n* (%)	31 (19.0)	157 (17.9)	0.729
Diuretics use, *n* (%)	8 (4.9)	52 (5.9)	0.632
Nondiuretic antihypertensives use, *n* (%)	63 (38.7)	343 (38.6)	0.995
Sleep medication use, *n* (%)	18 (11.0)	84 (9.5)	0.536
Depressive symptoms^a^, *n* (%)	31 (19.0)	125 (14.1)	0.119
Physical activity, MET-minutes/week, median (IQR)	1,188 (330–2,613)	1,371 (495–2,772)	0.190
Environmental variables			
Daytime outdoor temperature, mean (SD), °C	8.7 (4.3)	8.5 (5.0)	0.653
Daytime indoor temperature, mean (SD), °C	15.3 (4.3)	15.8 (3.8)	0.144
Day length, minutes, median (IQR)	641 (613–674)	656 (623–684)	0.023
Variables on nocturnal urination			
Nocturnal urine volume, mL, mean (SD)	563.3 (269.6)	570.7 (254.3)	0.745
Nocturnal urine production rate, mL/hour, mean (SD)	66.8 (33.8)	70.7 (31.5)	0.152
Single voided urine volume, mL, median (IQR)^b^	250 (173–330)	282 (205–380)	0.004
Night-time frequency ≥2 voids, *n* (%)	66 (40.5)	242 (27.3)	0.001

eGFR, estimated glomerular filtration rate; JPY, Japanese yen; SD, standard deviation.

*P*-values were calculated using the unpaired *t*-test and χ^2^ test.

^a^15-item Geriatric Depression Scale ≥6.

^b^Tested after natural log-transformation.

### Hot-water bathing and nocturia

Compared with the no bathing group, the bathing group was significantly associated with a lower prevalence of nocturia (Table 2). The OR of nocturia between the bathing and no bathing groups was 0.68 (95% confidence interval [CI], 0.48–0.97; *P* = 0.035) after adjusting for basic variables (age, sex, obesity, current smoking, alcohol consumption, and household income), clinical variables (diabetes mellitus, eGFR, medication use [diuretics, nondiuretic antihypertensives, and hypnotics], depressive symptoms, and physical activity [per quartile change]), and environmental variables (daytime indoor and outdoor temperatures, and day length [per quartile change]) (model 3 in Table 2). The single voided urine volume of the bathing group was higher than that of no bathing group after adjusting for basic, clinical, and environmental variables (mean difference: 0.083 log-mL; 95% CI, 0.004–0.163; *P* = 0.040).

**Table 2.** Odds ratio of nocturia associated with hot-water bathing

	Hot-water bathing		*P* value

	without (*n* = 163)	with (*n* = 888)	
Unadjusted OR (95% CI)	1.00 (Ref.)	0.55 (0.39–0.78)	0.001

Adjusted OR (95% CI)			
Model 1^a^	1.00 (Ref.)	0.67 (0.47–0.94)	0.019
Model 2^b^	1.00 (Ref.)	0.67 (0.47–0.95)	0.025
Model 3^c^	1.00 (Ref.)	0.68 (0.48–0.97)	0.035

CI, confidence interval; OR, odds ratio; Ref., reference.

ORs of nocturia were calculated using the logistic regression model.

^a^Adjusted for age, sex, and body mass index (≥25 kg/m^2^).

^b^Adjusted for current smoking, alcohol consumption (≥30 g/day), household income (≥4 million Japanese yen), diabetes mellitus, estimated glomerular filtration rate (<60 mL/min/1.73 m^2^), medication use (diuretics, nondiuretic antihypertensives, hypnotics), depressive symptoms (15-item Geriatric Depression scale ≥6), physical activity (per quartile change), in addition to Model 1.

^c^Adjusted for daytime outdoor and indoor temperatures, and day length (per quartile change), in addition to Model 2.

### Bathing conditions and nocturia

A higher night-time urinary frequency was significantly associated with later bathing time, longer bath-to-bed interval, later bedtime, and longer time in bed (Table 3). The Q2 (61–100 minute) and Q3 (101–160 minute) groups of bath-to-bed interval were significantly associated with a lower prevalence of nocturia than the Q4 (161–576 minute) group (Table 4). After adjusting for basic, clinical, and environmental variables, in addition to bathing duration, hot-water temperature, bedtime, and time in bed, the associations remained significant (adjusted OR in Table 4). By contrast, bathing duration and hot-water temperature were not significantly associated with the prevalence of nocturia in the adjusted model.

**Table 3.** Bathing and sleep parameters stratified by night-time frequency

	All	Night-time frequency (voids/night)				*P* trend

		0	1	2	≥3	
No. of participants	888	250	396	177	65	
Bathing parameters, mean (SD)						
Start time of bathing	20:34 (1:48)	21:07 (1:45)	20:36 (1:38)	19:58 (1:59)	19:46 (1:34)	<0.001
End time of bathing	21:02 (1:48)	21:35 (1:45)	21:04 (1:45)	20:26 (1:59)	20:13 (1:36)	<0.001
Bath-to-bed interval, min^a^	100.0 (60.0–160.0)	93.5 (60–150)	102 (61–158)	108 (60–177.5)	115 (65–195)	0.044
Bathing duration, min	13.0 (6.9)	12.9 (7.5)	12.6 (6.2)	13.6 (6.3)	14.1 (9.5)	0.109
Hot water temperature, °C	40.73 (1.36)	40.72 (1.36)	40.72 (1.39)	40.73 (1.30)	40.80 (1.35)	0.720
Bath room temperature, °C	16.4 (4.3)	16.7 (4.5)	16.4 (4.1)	15.8 (4.4)	16.3 (4.6)	0.126
Dressing room temperature, °C	13.4 (4.1)	13.5 (4.0)	13.5 (4.0)	13.1 (4.3)	13.6 (4.3)	0.622
Sleep parameters, mean (SD)						
Bedtime	22:37 (1:12)	23:04 (1:12)	22:37 (1:07)	22:11 (1:11)	21:55 (1:05)	<0.001
Rising time	6:47 (0:57)	6:38 (0:59)	6:48 (0:55)	6:50 (0:59)	7:01 (0:50)	<0.001
Time in bed, min	490.9 (80.5)	455.0 (80.5)	491.6 (72.7)	519.7 (73.7)	546.8 (80.3)	<0.001

Bath-to-bed interval, interval from start time of bathing to bedtime; SD, standard deviation.

*P*-values for trend were calculated using the linear regression model.

^a^Expressed as median (interquartile ranges) and tested after natural log transformation.

**Table 4.** Odds ratios for nocturia according to changes in bathing and sleep variables (*n* = 888)

	Unadjusted OR	*P* value	Adjusted OR^a^	*P* value
	(95% CI)		(95% CI)	
Bathing parameters				
Bath-to-bed interval				
Q1 [10–60 min] vs Q4	0.77 (0.51–1.15)	0.202	0.73 (0.47–1.15)	0.178
Q2 [61–100 min] vs Q4	0.62 (0.41–0.94)	0.026	0.60 (0.38–0.96)	0.031
Q3 [101–160 min] vs Q4	0.58 (0.38–0.89)	0.012	0.59 (0.37–0.94)	0.025
Q4 [161–576 min]	1.00 (Ref.)	—	1.00 (Ref.)	—
Bathing duration (per 1 min)	1.02 (1.00–1.04)	0.047	1.01 (0.99–1.04)	0.320
Hot water temperature (per 1°C)	1.02 (0.92–1.14)	0.681	0.97 (0.86–1.09)	0.607

Sleep parameters				
Bedtime (per 1 hour later)	0.62 (0.55–0.70)	<0.001	0.81 (0.67–0.99)	0.043
Time in bed (per 1 hour)	1.62 (1.46–1.80)	<0.001	1.24 (1.03–1.49)	0.024

Bath-to-bed interval, interval from start time of bathing to bedtime; CI, confidence interval; OR, odds ratio; Ref. reference.

^a^Adjusted for age, sex, obesity (body mass index ≥25 kg/m^2^), current smoking, alcohol consumption (≥30 g/day), household income (≥4 million Japanese yen), diabetes mellitus, estimated glomerular filtration rate (<60 mL/min/1.73 m^2^), medication use (diuretics, nondiuretic antihypertensives, hypnotics), depressive symptoms (15-item Geriatric Depression scale ≥6), physical activity (per quartile change), outdoor and indoor temperatures, day length (per quartile change), and all variables shown in this Table.

ORs of nocturia were calculated using the logistic regression model, which expresses the ORs to the reference group or the ORs per 1.0 unit increase in parameters.

In the Poisson regression analysis, night-time frequency in the Q2 and Q3 groups of bath-to-bed interval were significantly lower than those in the Q4 group (eTable 1). The ratios of night-time frequency (the ratios of the Q1/Q4, Q2/Q4 and Q3/Q4 group of bath-to-bed interval) were 0.90 (95% CI, 0.78–1.04; *P* = 0.163), 0.82 (95% CI, 0.71–0.96; *P* = 0.013), and 0.86 (95% CI, 0.74–0.99; *P* = 0.048), respectively, after adjusting for basic, clinical, environmental variables, in addition to bathing duration, hot-water temperature, bedtime and time in bed (adjusted nocturnal voids ratio in eTable 1). By contrast, there was no significant association of bathing duration and hot-water temperature with night-time frequency in the adjusted model.

## DISCUSSION

In this study, PBH via hot-water bathing was associated with a lower prevalence of nocturia in the cold environment, independent of potential confounders, such as age, sex, obesity, medication use, physical activity, and indoor temperature. Hot-water bathing with a bath-to-bed interval of 61–160 minutes was significantly associated with a lower prevalence of nocturia and night-time frequency than a bath-to-bed interval of ≥161 minutes. Furthermore, single voided urine volume, a surrogate for functional bladder capacity, was significantly higher in the bathing group. However, nocturnal urine volume and production rate were not significantly different between the two groups. To our knowledge, this is the first study that evaluated an association between PBH via hot-water bathing and nocturia.

Although the mechanisms underlying the association between PBH and nocturia remain uncertain, previous studies showed that improvement in cold-induced lower urinary tract symptoms (LUTS) with medication and exercise was associated with high skin temperature and low cold sensitivity.^22^^,^^23^ A galenical solution extracted from traditional herbal medicine increased skin temperature and reduced cold-induced detrusor overactivity in rats.^22^ Administration of the solution reduced the expression of transient receptor potential cation channel subfamily melastin member 8, an ion channel expressed in cold-sensing neurons,^23^ in the skin tissues of rats. An exercise intervention conducted in the cold season increased skin temperature and reduced LUTS in individuals with cold sensitivity.^22^ Our previous study showed that hot-water bathing with a bath-to-bed interval of 61–180 minutes was associated with a high skin temperature at bedtime.^11^ The bath-to-bed interval was similar to those of the Q2 and Q3 groups in the present study. In the present study, the single voided urine volume was larger in the bathing group, suggesting that the before-bedtime PBH is associated with a larger functional bladder capacity.

Possible explanations for the association of PBH with nocturia include decreased nocturnal urinary volume, improvement in sleep quality, and circadian rhythm modification. Previous studies have showed that hot-water bathing with optimal timing was associated with lower nighttime blood pressure, shorter sleep onset latency, and better sleep efficiency.^11^^–^^13^ An interventional study in patients with insomnia showed ramelteon alleviated insomnia, concurrent with a decrease in the number of nocturnal voids and an increase in nighttime bladder capacity,^24^ suggesting interactions between sleep quality and nocturia. Moreover, previous RCTs revealed that repeated PBH via other forms improved chronic heart failure and depression.^25^^,^^26^ However, there was no significant difference in nocturnal urine volume or production rate between the bathing and no bathing groups in this study. One plausible explanation for this observation is that a change in the body weight after a single session of hot-water bathing, which ranged 71–130 g depending on the manner of hot-water bathing,^27^ could be compensated by fluid intake after bathing because this study was non-interventional. Changes in body temperature caused by environmental temperature might modify the circadian rhythm of peripheral organs, including the kidney and bladder,^28^ because circadian oscillations of peripheral tissues are sensitive to temperature changes within the physiological temperature range (36.0–38.5°C).^29^

Our results implied that the estimated reduction in night-time frequency in correlation with PBH might not be lower than that induced by medications for benign prostatic hyperplasia (BPH) and overactive bladder (OAB). Based on our findings regarding the night-time frequency ratio Q2/Q4 group of bath-to-bed interval in the adjusted model (0.82; 95% CI, 0.70–0.96), the hypothetical reductions of night-time frequency in the Q2 group were 0.36 (2 − 0.82 × 2), 0.54 (3 − 0.82 × 3), and 0.72 (4 − 0.82 × 4) voids, where night-time frequency were 2, 3, and 4 voids in the Q4 group, respectively. In previous RCTs of patients with BPH, the night-time frequency among patients treated with doxazosin and dutasteride were reduced by 0.16 and <0.30 voids compared with placebo, respectively.^30^^,^^31^ Similarly, the night-time frequency among patients with OAB treated with trospium chloride, which is the most effective antimuscarinic for nocturia, was reduced by 0.24 voids compared with placebo.^32^

The strengths of this study include the use of real-world data obtained via the detailed measurements of the indoor environment and inclusion of a large sample, which enabled multivariable analysis with clinical and environmental variables.

This study has several limitations. First, the participants were not randomly selected, which could lead to selection bias. However, some results, including BMI and eGFR, were similar to nationwide data.^33^ Second, residual confounding was possible because we did not confirm whether BPH, OAB, peripheral edema, and sleep apnea were present and did not measure fluid intake and 24-hour urine volume. Third, night-time frequency was examined only for one night. The measurements may not be representative data. However, we reported the moderate reproducibility of night-time frequency in 189 participants who underwent additional assessments of night-time frequency (κ coefficient = 0.55).^34^ Fourth, core body temperatures were not measured. Therefore, further studies should be performed to assess heat load, heat loss, and temperature rhythm.

PBH with hot-water bathing was associated with a lower prevalence of nocturia among community-dwelling older adults. A bath-to-bed interval of 61–160 minutes was significantly associated with a low prevalence of nocturia and low night-time frequency. These findings can provide a framework for future interventional studies examining the effect of PBH on nocturia.

## SUPPLEMENTARY MATERIAL

eTable 1. Ratio of night-time frequency according to changes in bathing and sleep parameters (n = 888)
