# Supplementary material for: Association Between Before-bedtime Passive Body Heating and Nocturia During the Cold Season Among Older Adults
Source: J Epidemiol. 2023 Aug 5;33(8):398–404. doi: 10.2188/jea.JE20210471 (PMC10319524; doi:10.2188/jea.JE20210471)
Supplement: Supplementary file 1 [file je-33-398-s001.pdf]

eTable 1. Ratio of night-time frequency according to changes in bathing and sleep parameters (n=888)

	Nocturnal voids ratio Unadjusted (95% CI)	*P* value	Nocturnal voids ratio Adjusted[a] (95% CI)	*P* value
Bathing parameters				
Interval from bathing to bedtime				
Q1 [10–60 min] vs. Q4	0.89 (0.76, 1.04)	0.131	0.90 (0.78, 1.04)	0.163
Q2 [61–100 min] vs. Q4	0.80 (0.68, 0.95)	0.009	0.82 (0.71, 0.96)	0.013
Q3 [101–160 min] vs. Q4	0.83 (0.71, 0.97)	0.018	0.86 (0.74, 0.99)	0.048
Q4 [161–576 min]	1.00 (Ref.)	–	1.00 (Ref.)	–
Bathing duration (per 1 min)	1.01 (1.00, 1.02)	0.048	1.00 (0.99, 1.01)	0.434
Hot water temperature (per 1 °C)	1.01 (0.97, 1.06)	0.574	1.00 (0.96, 1.04)	0.929
Sleep parameters				
Bedtime (per 1 hour later)	0.82 (0.79, 0.85)	<0.001	0.94 (0.89, 1.01)	0.111
Time in bed (per 1 hour)	1.22 (1.18, 1.26)	<0.001	1.12 (1.05, 1.19)	<0.001

CI, confidence interval.

Ratios of night-time frequency were calculated using the Poisson regression model, which expresses the ratios to the reference group or the ratios per 1.0 unit increase in parameters.

[a]Adjusted for age, sex, obesity (BMI ≥25 kg/m²), current smoking, alcohol consumption (≥30 g /day), household income (≥4 million JPY), diabetes mellitus, eGFR (<60 mL/min/1.73 m²), medication use (diuretics, nondiuretic antihypertensives, hypnotics), depressive symptoms (15-item Geriatric Depression scale ≥6), physical activity (per quartile change), outdoor and indoor temperatures, day length (per quartile change), and all variables shown in this Table.
